# A unified analytical framework for Mössbauer synchrotron sources

**DOI:** 10.1107/S1600577526005357

**Published:** 2026-06-19

**Authors:** Krzysztof R. Szymański

**Affiliations:** a Faculty of Physics University of Bialystok K. Ciolkowskiego 1L 15-245Bialystok Poland; Bhabha Atomic Research Centre, India

**Keywords:** synchrotron Mössbauer source, X-ray free-electron lasers, hyperfine interactions, intensity tensor formalism, Fisher information analysis

## Abstract

Next-generation Mössbauer spectroscopy at synchrotron and X-ray free-electron laser facilities demands rapid, accurate and polarization-aware modeling of nuclear hyperfine interactions. We present a unified analytical framework that provides exact, rotationally invariant expressions for resonance energies and transition probabilities in the presence of simultaneous magnetic dipole and electric quadrupole interactions. Unlike conventional approaches, our method avoids Hamiltonian diagonalization by expressing intensities entirely in terms of hyperfine invariants, enabling efficient global fitting and modeling of hyperfine-interaction distributions in complex materials. We further introduce a quantitative identifiability metric and demonstrate, via Monte Carlo sampling, that polarization control—particularly orthogonal linear polarizations—substantially improves hyperfine-parameter determination. This work offers a mathematically transparent and computationally efficient toolset for modern Mössbauer spectroscopy, accelerating studies of iron-based compounds and magnetic, electronic and structural order under extreme conditions and at nanoscale geometries.

## Abstract 2

We present a unified analytical framework for Mössbauer synchrotron and X-ray free-electron laser sources that provides exact, rotationally invariant expressions for resonance energies and transition probabilities in mixed magnetic dipole–electric quadrupole interactions without Hamiltonian diagonalization. The invariant formalism enables efficient modeling and quantitative identifiability analysis, demonstrating that polarization control—especially orthogonal linear polarizations—significantly enhances the determination of hyperfine parameters in complex materials.

## 1. Introduction

The discovery of recoilless nuclear resonance fluorescence by Rudolf Mössbauer in 1958 revolutionized spectroscopy by enabling the observation of nuclear transitions with an energy resolution exceeding 10^−13^. This extraordinary precision opened direct access to hyperfine interactions—the coupling of nuclear moments with their electronic and structural environment—thereby establishing a link between atomic-scale magnetism, chemical bonding and lattice symmetry. Since then, Mössbauer spectroscopy has become a cornerstone technique across condensed matter physics, chemistry, materials science and geophysics (Gütlich *et al.*, 2011 ▸).

Conventional Mössbauer spectroscopy relies on radioactive sources of limited intensity. The advent of synchrotron radiation has enabled time-domain nuclear resonant scattering (Gerdau *et al.*, 1986 ▸; Rüffer, 2008 ▸), while the subsequent development of synchrotron Mössbauer sources (SMS) transformed the method into a tunable, quasi-monochromatic energy-domain technique with polarized radiation (Mitsui *et al.*, 2015 ▸; Yaroslavtsev & Chumakov, 2022 ▸). The SMS technique allows quantitative investigations of microscopic samples with very small size, thin surfaces, and materials subjected to extreme pressure and temperature conditions (Mitsui *et al.*, 2009 ▸; Potapkin *et al.*, 2012 ▸; Fujiwara *et al.*, 2024 ▸). More recently, experiments at X-ray free-electron lasers (XFELs) have extended the scope of Mössbauer spectroscopy to single-pulse and nonlinear regimes (Chen *et al.*, 2022 ▸; Lentrodt *et al.*, 2025 ▸). This is particularly relevant for modern synchrotron and XFEL experiments, where controlled polarization states and time-gated detection enable direct access to otherwise inaccessible hyperfine parameters and their correlations.

The interpretation of Mössbauer spectra requires accurate evaluation of transition probabilities derived from the squared matrix elements of the transition operator. When magnetic dipole and electric quadrupole interactions are both present, this analysis becomes intricate due to the strong coupling of nuclear spin states and their dependence on multiple tensor orientations. The intensity tensor formalism, originally proposed by Zimmermann (1975 ▸), developed by Spiering (1977 ▸) and later by Szymański (2000 ▸, 2006 ▸), offers an elegant framework for describing transition intensities without explicit determination of eigenstates. However, previous invariant-based formulations were developed primarily for unpolarized or circularly polarized radiation and often relied on specific parametrizations of the hyperfine Hamiltonian that become ill-defined in limiting cases of vanishing interaction strengths. As a consequence, their applicability to modern SMS and X-ray free-electron laser (XFEL) experiments—where polarization control plays a central role—remains limited.

In this work, we present a unified analytical framework that overcomes these limitations. The approach is based on a separation of magnetic dipole and electric quadrupole interactions and on a consistent construction of rotational invariants that remain well defined over the entire parameter space. Within this formulation, the intensity-tensor formalism is extended to encompass arbitrary polarization states, including linear polarization, which introduces additional observable sensitivity to hyperfine parameters.

A key result is that all polarization cases—linear, circular and unpolarized—can be described within a single, compact tensor formalism, eliminating the need for separate treatments. This provides a consistent and practically applicable framework for the analysis of polarization-resolved Mössbauer spectra.

Furthermore, we address the problem of parameter identifiability by introducing a quantitative metric based on Fisher information. This analysis demonstrates that polarization control, particularly the use of orthogonal linear polarization states, significantly enhances the robustness and uniqueness of hyperfine-parameter determination.

## 2. Magnetic dipole and electric quadrupole interactions of nuclear spin

**Figure 1:**
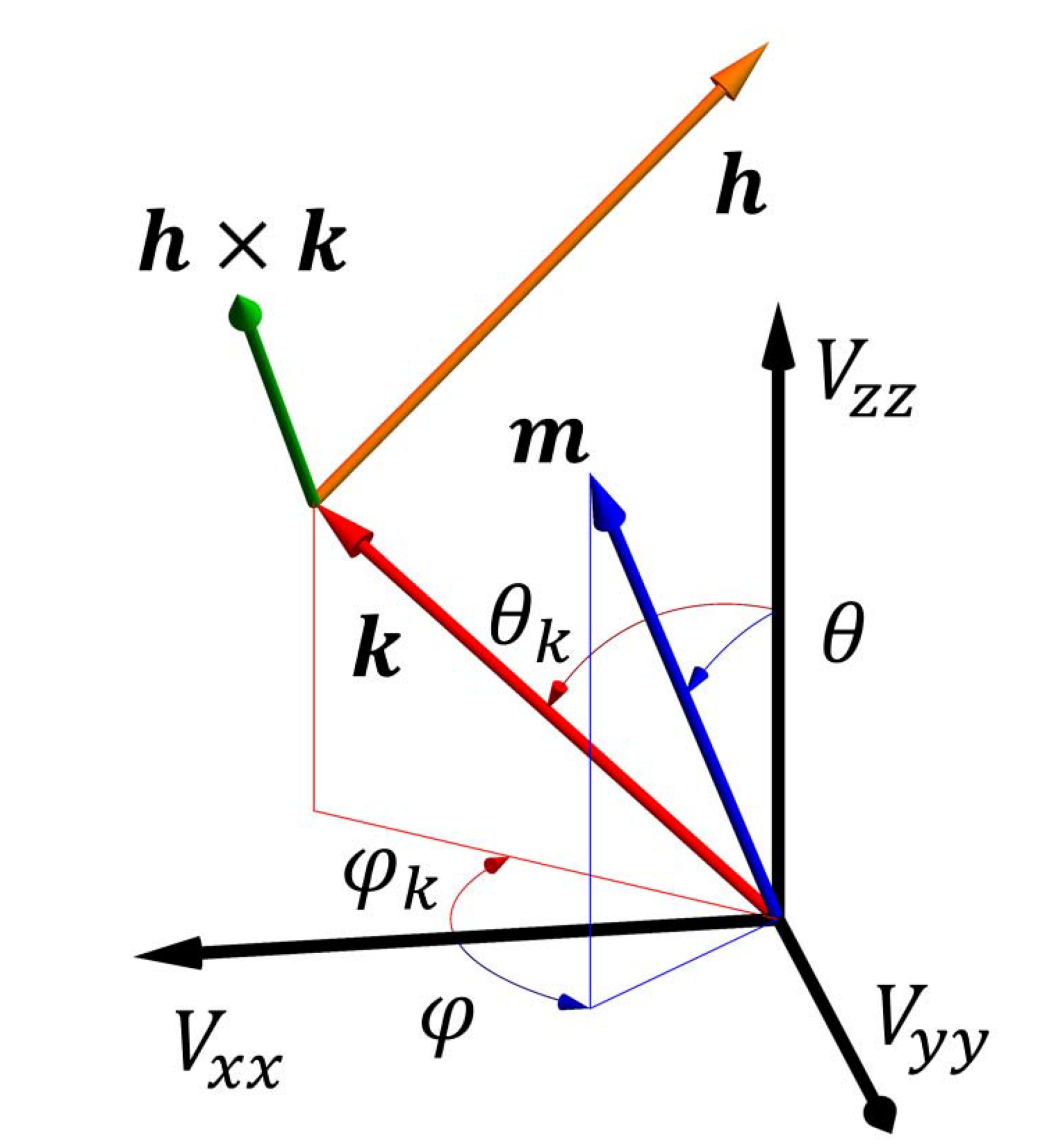
Directions of unit vectors **m** = **B**/|**B**|, photon wavevector **k**, and magnetic polarization of linearly polarized photon **h** in the PAS of the EFG.

Static interactions of the electronic environment with nuclear spin **I** consist of the coupling of nuclear magnetic moment **μ** = *g*μ_N_**I** with an effective magnetic field **B** and of nuclear quadrupole moment *Q* with the electric field gradient (EFG) tensor **V** with Cartesian components *V*_*ij*_. The EFG in velocity units is defined as
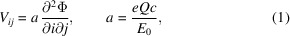
where Φ is the electric potential, *e* the elementary charge, *c* the speed of light and *E*_0_ the transition energy. The EFG tensor is traceless, symmetric and diagonal in its principal axis system (PAS),
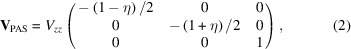
with asymmetry parameter
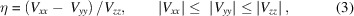
satisfying 0 ≤ η ≤ 1. The nuclear spin Hamiltonian including magnetic dipole and electric quadrupole interactions is (Brown & Parker, 1955 ▸; Matthias *et al.*, 1962 ▸; Kündig, 1967 ▸)
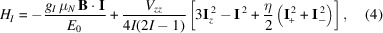
where 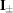 = **I**_*x*_ ± *i***I**_*y*_. For nuclear transitions between spin states *I* = 3/2 and *I* = 1/2 the Hamiltonians in the |*I*, *m*〉 basis take explicit forms, with energies expressed in velocity units and angles shown in Fig. 1 ▸ (see also Tables 1 ▸ and 2 ▸).
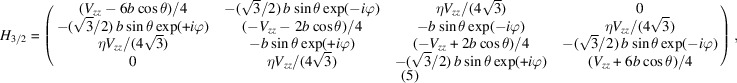

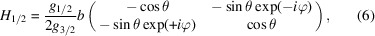
with
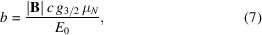
where *g*_*I*_ are nuclear *g*-factors for spin states *I* = 3/2, 1/2.

## 3. Rotational invariants

The explicit form of the Hamiltonian depends on the choice of the Cartesian coordinate frame, which often introduces technical and algebraic inconveniences. To circumvent these issues, we employ frame-independent rotational invariants, ensuring that all derived quantities—such as absorption line positions and intensities—are independent of the specific frame chosen.

For the following derivations, we define rotational invariants associated with tensor **V** and the hyperfine magnetic field direction **m** = **B**/|**B**|,
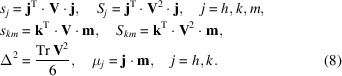
Here, lower case *s* refers to invariants of **V**, uppercase *S* to those of **V**^2^; **k** is the photon wavevector (any incident photon), and **h** the magnetic polarization vector for linear polarization (other cases are treated separately). The parameter Δ = |*V*_*zz*_/2|(1 + 1/3η^2^)^1/2^ corresponds to the quadrupole splitting, *i.e.* the separation of the doublet when *B* = 0.

## 4. Hamiltonian energies and states

The excited state energies 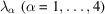 of *H*_3/2_ satisfy the quartic equation
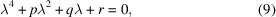
with coefficients expressed first by general relations with condition Tr*H*_3/2_ = 0 and after using the parameters hyperfine ineractions by invariants (8), and finally entirely in terms of invariants
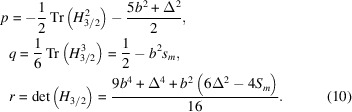
The ground state energies are ±*bg*_1/2_/(2*g*_3/2_). The solutions for 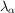 are presented in Appendix *A*[App appa], whereas an analysis of the (*p*, *q*, *r*) manifold accessible through physically allowed hyperfine interaction parameters is provided in Appendices *B*[App appb] and *C*[App appc]. The excited and ground eigenstates are abbreviated, respectively, as
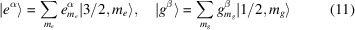
with β = ±1.

## 5. Intensity tensor

The *M*th component of the spherical amplitude for the transition αβ is given by
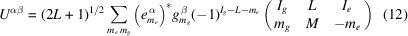
with *L* = 1, *I*_*g*_ = 1/2, *I*_*e*_ = 3/2. The spherical intensity tensor is
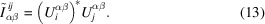
Transformation to Cartesian components yields the symmetric tensor
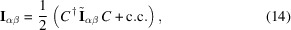
and the asymmetric part as a pseudovector
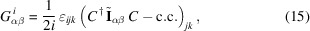
with transformation matrix
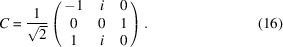
Although the intensity tensor is constructed from the eigenstates of the Hamiltonian, it was shown by Szymański (2000 ▸) that its components can instead be expressed solely in terms of the excited energies 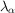 and the hyperfine interaction parameters—the hyperfine magnetic field and the EFG. Thus, the formulation does not require explicit eigenvectors; the tensor is written entirely through energy eigenvalues and hyperfine invariants. The explicit invariant-based forms are
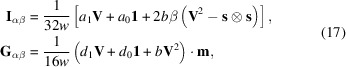
with denominator
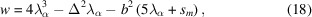
vector
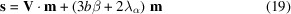
and coefficients
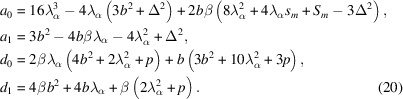
Operator ⊗ in (17) denotes the tensor product, *i.e.* an operation that combines two vectors into a second-order tensor.

## 6. Probabilities and energies of transition

After lengthy but exact algebra, we obtain closed invariant-based expressions for the eight transition probabilities *i*_αβ_, α = 1,…, 4, β = ±1, normalized to unity. Each probability is a rational function of the excitation energies 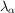 with coefficients depending solely on the previously introduced invariants and the auxiliary denominator *w*. For unpolarized radiation,
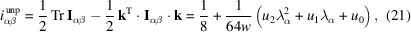
with
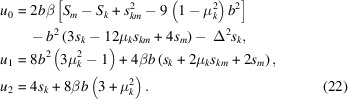
For circularly polarized radiation
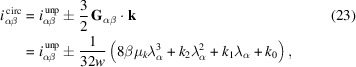
where signs ± corresponds to two circular polarizations and
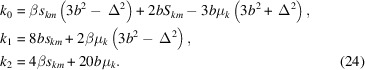
For linearly polarized radiation with magnetic polarization vector **h**,
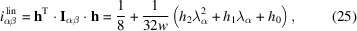
with
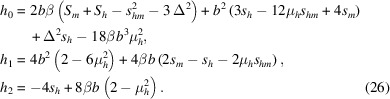
For texture free spectrum,
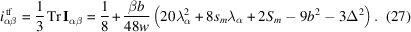
The line positions *v*_αβ_ are identical for all cases and given by
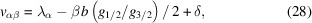
where δ is the isomer shift, omitted for brevity until now. We recall that, throughout the manuscript, we adopt a consistent notation: the parameter α serves solely as an index and takes the values α = 1,…, 4, while the parameter β takes the values β = ±1.

Finally, to improve clarity and accessibility for readers, a simplified step-by-step algorithmic outline for calculating line positions and transition probabilities is presented. To apply the model for spectral fitting and determination of the physical parameters *B*, *V*_*zz*_, η, θ, φ, θ_*k*_, φ_*k*_ and θ_*h*_, φ_*h*_, one first constructs the vector **m**(θ, φ) = (sinθcosφ, sinθsinφ, cosθ). Next, (2) is used to determine **V**. The invariants *s*_*m*_, *S*_*m*_ and Δ are then calculated using (8). Subsequently, the coefficients (*p*, *q*, *r*) are obtained from (10), the quartic equation (9) is solved with the aid of Appendix *A*[App appa], and the absorption line positions are determined from (28).

To calculate transition probabilities, one first constructs the photon propagation vector **k**(θ_*k*_, φ_*k*_) and the magnetic polarization vector **h**(θ_*h*_, φ_*h*_). The invariants *s*_*k*_, *S*_*k*_, *s*_*h*_, *S*_*h*_*s*_*km*_, *S*_*km*_ and μ_*h*_, μ_*jk*_ are then calculated using (8). The normalization denominator *w* is evaluated from (18). Finally, the transition probabilities are calculated according to the relevant case of photon polarization or texture using (21), (23), (25) or (27). Symbols with their physical meaning are summarised in Table 3 ▸.

## 7. Analysis of parameter estimation

We consider the problem of estimating hyperfine parameters of a single-site Mössbauer absorber with an unknown fixed orientation under the assumption that both the resonance velocities and line intensities are known.

It should be noted that the Fisher information analysis is based on idealized assumptions of uncorrelated data with uniform variance and independently known resonance velocities and line intensities. In real Mössbauer experiments, correlations between spectral parameters, finite background and instrumental effects may reduce the effective information content. Therefore, the present results should be interpreted primarily in a qualitative sense when comparing different polarization schemes.

Let 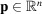 denote the parameter vector and 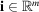 the measured data (*m* ≥ *n*) related by a nonlinear model **i**(**p**). For uncorrelated data with equal variance σ^2^, the Fisher information matrix (van den Bos, 2011 ▸) is
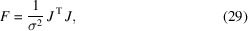
where 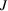 = 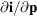

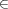

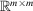
 is the Jacobian. The matrix *F* is symmetric with ordered eigenvalues
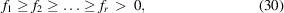
where *r* = rank(*F*) 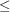
*n* gives the number of identifiable linear parameter combinations.

As a compact measure of the numerical conditioning and parameter identifiability, we define the invertibility index
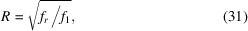
which is independent of the noise variance σ^2^. Higher *R* values indicate better-conditioned parameter estimation.

Monte Carlo simulations were performed to obtain the distribution of the invertibility index *R*. The parameters *B*, *V*_*zz*_, η, θ, φ, θ_*k*_, φ_*k*_, θ_*h*_, φ_*h*_ were randomly sampled from their physical domains under the constraint 1 < *B*/*V*_*zz*_ < 5 (in T s mm^−1^), ensuring that the resulting spectra are neither close to the pure Zeeman sextet nor to the quadrupole doublet limit. The isomer shift was not included. The resulting *R* distributions exhibit asymmetric peaks characterized by the position of the maximum μ. For the probability level corresponding to one standard deviation (∼68%), the highest-probability interval of minimal width was selected, and its distances from μ define asymmetric deviations σ_*L*_ and σ_*R*_ (Feldman & Cousins, 1998 ▸). The minimum and maximum values, *R*_min_ and *R*_max_, observed in the simulations are also reported (Table 4 ▸).

## 8. Discussion

Equations (21)–(27) are fully consistent with results obtained by direct diagonalization of the Hamiltonian and evaluation of squared transition matrix elements, in agreement with earlier studies (Brown & Parker, 1955 ▸; Matthias *et al.*, 1962 ▸; Kündig, 1967 ▸).

**Figure 2:**
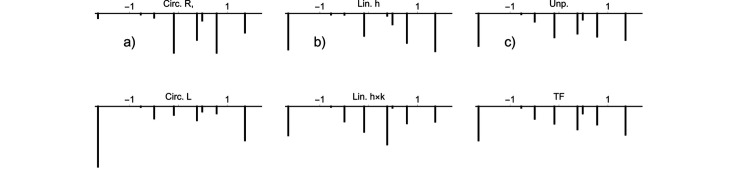
Schematic representation of line positions and transition probabilities for the parameters discussed in the text: (*a*) circular polarization states (top and bottom), (*b*) linear polarization with magnetic polarization vector **h** (top) and the orthogonal linear polarization state with magnetic polarization vector along **h** × **k** (bottom), (*c*) unpolarized radiation with **k** direction (top) and texture free spectrum (bottom). Horizontal scale is in mm s^−1^.

**Figure 3:**
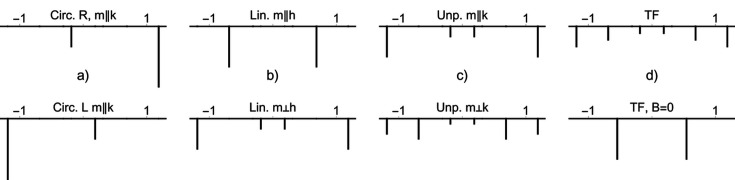
Graphic presentation of line positions and transition probabilities for (*a*) wavevector **k** parallel to **m** for two opposite circular polarizations, (*b*) for linear polarization with **m** and **h** parallel (top) and perpendicular (bottom), (*c*) for unpolarized radiation with **m** and **k** parallel (top) and perpendicular (bottom), (*d*) for texture-free absorber with **V** = 0, *B* ≠ 0 (top) and **V** ≠ 0, *B* = 0 (bottom). The nonzero values of *B* and **V** correspond to those in the considered numerical example.

The principal significance of the derived explicit probabilities lies in their ability to model hyperfine-interaction distributions without Hamiltonian diagonalization. This analytic transparency is particularly valuable given the rapid evolution of computational platforms and the increasing difficulty of maintaining legacy Mössbauer software. The compact invariant expressions therefore meet a practical need for formulae that can be readily embedded in modern data-analysis environments. To support correct implementation, numerical examples are provided for the geometry in Fig. 1 ▸, with the corresponding line positions and intensities collected in Table 2 ▸ and visualized in Fig. 2 ▸. Some characteristic spectral shapes for representative orientations of the photon wavevector, hyperfine magnetic field and frequently used polarizations are illustrated schematically in Fig. 3 ▸.

Although the present work is theoretical, the derived expressions are directly applicable to realistic synchrotron Mössbauer spectroscopy data analysis, where they can be combined with standard line-broadening models and numerical fitting procedures. The observed improvement in parameter conditioning with linear polarization is expected to remain valid under typical experimental conditions. The present formulation is restricted to the thin-absorber limit and does not include dichroism and thickness effects, which may be relevant for linearly polarized radiation in thicker samples.

The explicit intensities further allow a rigorous treatment of the ambiguity problem (Karyagin, 1966 ▸; Satuła *et al.*, 2008 ▸). For unpolarized radiation, the first velocity moment corrected for the isomer shift is (Szymański, 2006 ▸)
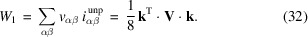
For texture-free spectra, *W*_1_ = 0. Since this condition cannot generally be fulfilled for arbitrary orientations of **k** and a general **V**, only special parameter sets admit a single-site representation that reproduces a texture-free spectrum.

The numerical parameter-identifiability analysis (Table 1 ▸) shows that all considered parameter sets are, in principle, determinable, as indicated by non-zero invertibility indices *R*. However, conditioning improves markedly when polarization information is included. Two orthogonal linear polarizations yield substantially better conditioning (μ = 0.1) than opposite circular polarizations (μ = 0.0054), reflecting the higher information content of linearly polarized radiation. The combination of circular and linear polarizations provides the best overall performance (μ = 0.112, *R*_max_ = 0.46). Nonetheless, small *R* values occur for certain configurations, indicating locally poor conditioning despite global identifiability—serving as a consideration for experiment design and data analysis.

Overall, the closed, rotationally invariant expressions presented here provide a unified and computationally efficient framework for modeling Mössbauer spectra with arbitrary polarization. Combined with the identifiability analysis, these results establish a rigorous foundation for quantitative interpretation in synchrotron and XFEL Mössbauer spectroscopy.

## 9. Summary

Compact, rotationally invariant algebraic expressions for Mössbauer transition probabilities and resonance line positions in the presence of simultaneous magnetic dipole and electric quadrupole interactions are derived. The formulas cover the most relevant polarization cases—linear, circular and unpolarized radiation—and express the intensities directly in terms of hyperfine field invariants, without requiring diagonalization of the spin Hamiltonian.

The invariant framework enables efficient implementation in computational tools and facilitates the modeling of hyperfine field distributions. A quantitative analysis of the invertibility of spectral components—absorption line positions and intensities—shows that the use of polarized radiation markedly improves the identifiability of hyperfine parameters, with linear polarization providing better conditioning than circular polarization.

The presented results establish a rigorous and practical foundation for modern synchrotron and XFEL Mössbauer spectroscopy, supporting quantitative interpretation and reliable modeling of hyperfine interactions. This is especially relevant for polarization-controlled synchrotron and XFEL experiments, where the proposed formalism directly supports advanced quantitative data analysis and experiment design.

## Supplementary Material

Zip file containing the Mathematica code. DOI: 10.1107/S1600577526005357/ye5085sup1.zip

## Figures and Tables

**Figure 4:**
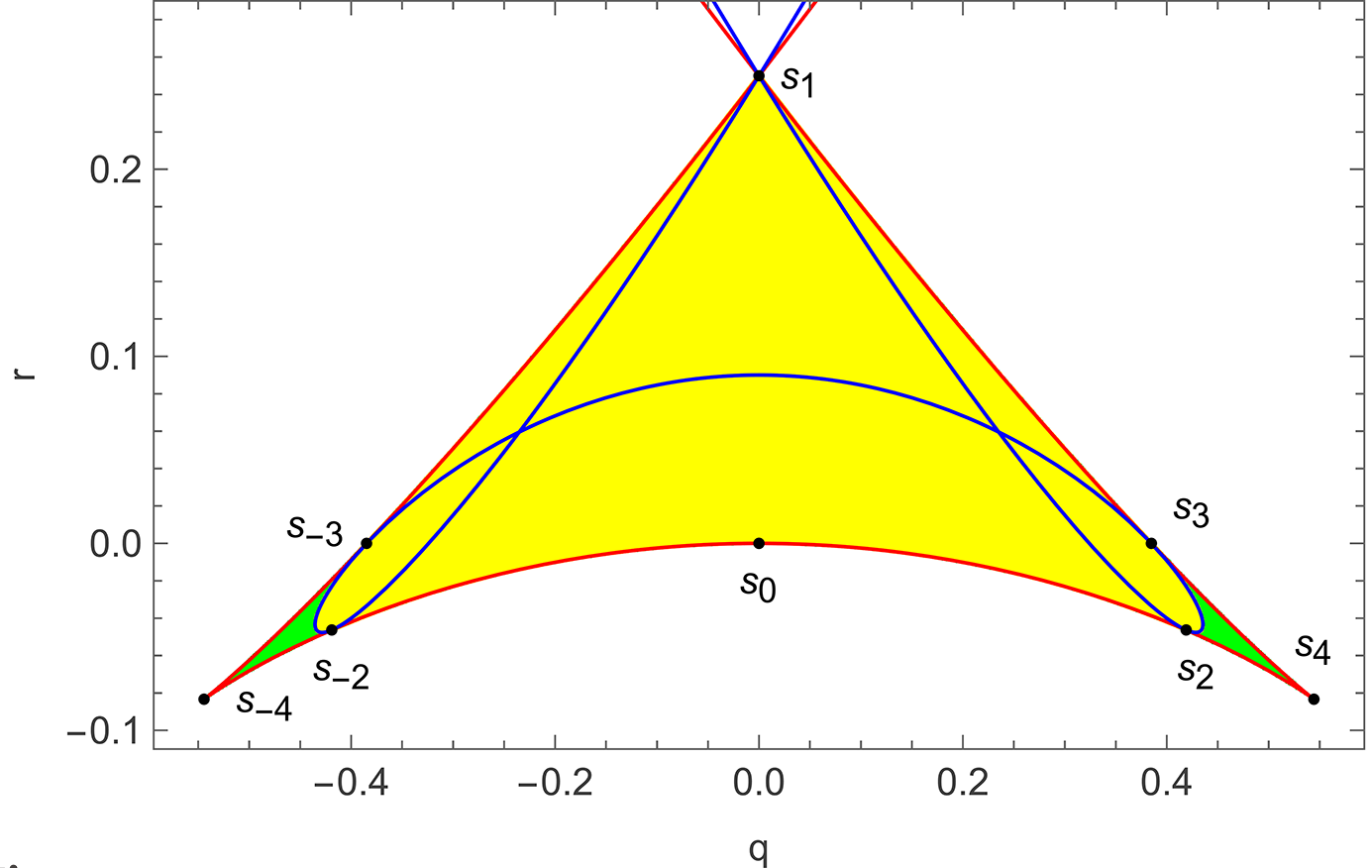
Zeros of *w*_1_ (red) and *w*_2_ (blue) for *p* = −1. Distinguished points *s*_*i*_ listed in Table 5 ▸ are indicated. Green points represent numerical solutions for which equation (9) possesses four real roots of, whereas the overlaid yellow points denote physically accessible hyperfine parameters.

**Table 1:** Polar and azimuthal angles of vectors **m**, **k**, **h** and **h**×**k** shown in Fig. 1 ▸

	Polar	Azimuthal
**m**	θ = π/6 = 0.524	φ = π/4 = 0.785
**k**	θ_*k*_ = π/3 = 1.047	φ_*k*_ = −π/4 = −0.785
**h**	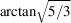 = 0.912	arccot 3 − π = −2.820
**h** × **k**	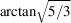 = 0.912	arctan 3 = 1.249

**Table 2:** Line positions and probabilities for the exemplary set of parameters considered in the text and Fig. 1 ▸

α, β	1, 1	1,−1	2, 1	3, 1	2,−1	4, 1	3,−1	4,−1
*v*_αβ_	−1.647	−0.772	−0.497	−0.094	0.378	0.487	0.781	1.362
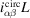	0.033	0.007	0.03	0.278	0.187	0.05	0.279	0.135
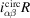	0.427	0.004	0.085	0.06	0.098	0.036	0.049	0.24
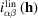	0.255	0.008	0.009	0.158	0.016	0.077	0.208	0.268
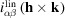	0.205	0.003	0.106	0.18	0.269	0.009	0.119	0.108
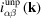	0.23	0.006	0.058	0.169	0.143	0.043	0.164	0.188
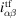	0.241	0.009	0.088	0.122	0.162	0.05	0.128	0.2

**Table 3:** Summary of symbols and invariants with their physical meaning

**V**, *V*_*ij*_	EFG expressed in velocity units
η	Asymmetry parameter of the EFG
*e*, *c*	Fundamental physical constants
*Q*	*I* = 3/2 nuclear quadrupole moment
*E*_0_	Energy of Mössbauer transition, 14.4 keV
*a*	Proportionality constant between EFG and velocity units, equation (1)
**B**	Effective magnetic field
**m**	Unit vector along effective magnetic field, in spherical coordinates **m** = (sinθcosφ, sinθsinφ, cosθ)
*b*	Dimensionless parameter proportional to |**B**|, equation (7)
*s*_*j*_	Invariant, scalar product of tensor **V** and unit vectors **j**, equation (8)
*S*_*j*_	invariant, scalar product of tensor **V**^2^ and unit vectors **j**, equation (8)
*s*_*km*_	Invariant, scalar product of tensor **V** and unit vectors **k** and **m**, equation (8)
*S*_*km*_	Invariant, scalar product of tensor **V**^2^ and unit vectors **k** and **m**, equation (8)
Δ	The separation of the doublet when *B* = 0, equation (8)
μ_*j*_	Scalar product of unit vectors **j** and **m**
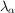	Eigenvalues of the Hamiltonian equation (5), α = 1,…, 4; roots of equation (9)
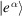 , 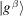	Excited and ground states, α = 1,…, 4, β = ±1
*p*, *q*, *r*	Invariants defined in equation (10), coefficients of secular equation (9)
**I**_αβ_	The intensity tensor
**G**_αβ_	Asymmetric part of the intensity tensor
**k**	Unit vector parallel to the photon wavevector
**h**	Magnetic polarization vector of linearly polarized photon
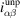	Intensities for unpolarized radiation, 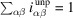 = 1
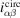	Intensities for circularly polarized radiation, 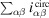
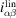	Intensities for linearly polarized radiation, 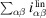 = 1
*v*_αβ_	Positions of absorption lines
δ	Isomer shift

**Table 4:** Statistical characteristics of the invertibility index *R* for different parameter sets and available information The symbols ± denote two opposite circular polarizations, while ∥ and 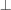 represent orthogonal linear polarizations (for magnetic polarizations along **h** and **h**×**k**).

	Parameters determined	Available information	μ	σ_*L*_	σ_*R*_	*R*_min_	*R*_max_
	1	2	3	4	5	6	7
1	*B*, *V*_*zz*_, η, θ, φ, θ_*k*_, φ_*k*_	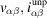	1.53 × 10^−3^	1.53 × 10^−3^	0.011	0	0.215
2		*v*_αβ_, ±	5.4 × 10^−3^	5.4 × 10^−3^	0.052	0	0.231
3		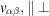	0.1	0.036	0.038	1.12 × 10^−3^	0.4
4		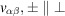	0.112	0.0284	0.067	1.53 × 10^−3^	0.46
5	*B*, *V*_*zz*_, η, θ, φ, θ_*k*_, φ_*k*_, θ_*h*_, φ_*h*_	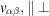	0.005	0.005	0.032	0	0.2
6		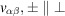	0.0071	7.1 × 10^−3^	0.056	2.94 × 10^−6^	0.221

**Table 5:** Distinguished points of the (*q*, *r*) manifold cross-section at fixed *p* < 0 and φ = π/2

Point	*q*	*r*	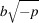	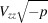	η	θ
*s*_0_	0	0	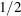	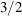	1	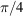
*s*_1_	0	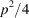	0	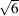	1	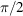
*s*_−2_, *s*_2_	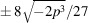	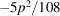	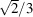	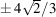	0	0
*s*_−3_, *s*_3_	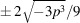	0	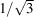	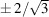	0	0
*s*_−4_, *s*_4_	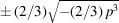	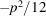	−	−	−	−

## Data Availability

The *Mathematica* source code is available as supplementary material.

## Appendix A Solution of the quartic equation

The solution to the quartic equation (9) is based on the classical method of L. Ferrari, with the associated resolvent cubic solved using the trigonometric approach of F. Viète. For improved computational efficiency, the auxiliary quantities are introduced in a form that is algebraically equivalent to the classical formulation but rearranged to reduce the number of arithmetic operations. The quartic equation is factorized into a product of two quadratic polynomials by introducing an auxiliary variable *X*, related to the resolvent cubic, expressed as

where

The four real roots are then given by

with

All introduced variables are real when all four roots are real. A similar formulation was reported by Häggström (1974 ▸), while the present form requires only a single evaluation of the trigonometric functions for all four roots.

## Appendix B Geometry of the physically accessible (*p*, *q*, *r*) manifold

We characterize the geometry of the (*p*, *q*, *r*) manifold defined in (10), generated by physically admissible hyperfine parameters: *b* ≥ 0, *V*_*zz*_ ∈ *R*, 0 ≤ η ≤ 1, 0 ≤ φ ≤ 2π, 0 ≤ θ ≤ π. Since *q* and *r* depend affinely on *s*_*m*_ and *S*_*m*_ (8), respectively, it is sufficient to consider only the two eigenspaces of **V** associated with extremal eigenvalues *V*_*zz*_ and −*V*_*zz*_(1 + η)/2 (Appendix *C*[App appc]). For the standard ordering of the diagonal components of **V**, this reduction corresponds to φ = π/2. The manifold is conveniently characterized by cross-sections at fixed *p*, using

For *p* ≤ 0, the mapping projects the three-dimensional parameter space (*V*_*zz*_, η, θ) onto a two-dimensional manifold in the (*q*, *r*) plane, where

with *z* = cos^2^θ. Candidate boundary curves are identified from the vanishing Jacobians

One solution is

yielding the implicit relation *w*_1_ = 0, where

see the red line in Fig. 4 ▸. Another solution is

yielding *w*_2_ = 0, where

see the blue line in Fig. 4 ▸. The use of *w*_1_ in the definition of *w*_2_ yields a compact algebraic representation. The two branches of *w*_1_ and *w*_2_ intersect at *s*_4_ and are tangent at *s*_±2_ and *s*_±3_. Points *s*_±4_ correspond to cone-like singularities of *w*_1_ (Fig. 4 ▸ and Table 5 ▸).

The zeros of *w*_1_ and *w*_2_ form candidate boundaries of the (*q*, *r*) manifold attainable by parameters of hyperfine interactions. Detailed analysis shows that the admissible (*q*, *r*) region is enclosed by curves connecting the points *s*_−3_, *s*_−2_, *s*_0_, *s*_2_, *s*_3_, *s*_1_, *s*_−3_. Notably, the regions enclosed by the paths *s*_−3_, *s*_−4_, *s*_−2_, *s*_−3_ and *s*_2_, *s*_3_, *s*_4_*s*_2_ are not attainable for physically admissible hyperfine parameters *b* ≥ 0, *V*_*zz*_ ∈ *R*, 0 ≤ η ≤ 1, 0 ≤ φ ≤ 2π, 0 ≤ θ ≤ π, although equation (9) still possesses four real roots for these (*q*, *r*) values, see the green regions in Fig. 4 ▸.

Monte Carlo sampling (10^6^ realizations) independently confirmed the analytically derived admissible region, with no points observed outside the predicted boundary (Fig. 4 ▸).

## Appendix C Lemma on eigenspaces associated with extremal eigenvalues

The characterization of admissible pairs (*s*_*m*_, *S*_*m*_) (8) will be done by reduction of the problem to the extremal eigenspaces, which follows from two elementary observations. For clarity of notation, in what follows we omit the index *mmm* in *s*_*m*_, *S*_*m*_, writing simply *s* and *S*. First, by the Cauchy–Schwarz inequality, every admissible pair satisfies the universal lower bound *S*.

Second, the squared components  of a unit vector define a probability distribution *p*_*i*_ ≥ 0, satisfying  = 1. Hence, the quantities *s* and *S* can be written as convex combinations of the spectral points , which lie on the convex function. It follows that the geometry of the achievable set is *determined by the chord connecting the extremal eigenvalues* λ_−_ and λ_+_. In particular, every achievable pair (*s*, *S*) can be realized by a vector supported only on the eigenspaces associated with λ_−_ and λ_+_.

Lemma 1 Let *V* = diag(λ_1_,…, λ_*N*_), with extremal eigenvalues λ_−_ < 0 < λ_+_. Define

for unit vectors *m* ∈ *R*^*N*^. Then every achievable pair (*s*, *S*) is attained by a vector *m* supported on the eigenspaces corresponding to λ_−_ and λ_+_.

Proof Let  = . Since  = 1 and *p*_*i*_ ≥ 0, we may write

Hence,

showing that the achievable set is precisely the convex hull of the spectral points . Since these points lie on the convex function *y* = *x*^2^, any point with prescribed first coordinate *s* ∈ [λ_−_, λ_+_] is attained on the chord joining  and . Equivalently, there exists *x* ∈ [0, 1] such that

Such a pair is attained by a vector supported entirely on the eigenspaces corresponding to λ_−_ and λ_+_. □
